# Parallel Tests of Whole Exome Sequencing and Copy Number Variant Sequencing Increase the Diagnosis Yields of Rare Pediatric Disorders

**DOI:** 10.3389/fgene.2020.00473

**Published:** 2020-06-11

**Authors:** Xuyun Hu, Ruolan Guo, Jun Guo, Zhan Qi, Wei Li, Chanjuan Hao

**Affiliations:** ^1^Beijing Key Laboratory for Genetics of Birth Defects, Beijing Pediatric Research Institute, Beijing Children's Hospital, Capital Medical University, National Center for Children's Health, Beijing, China; ^2^MOE Key Laboratory of Major Diseases in Children, Beijing Children's Hospital, Capital Medical University, National Center for Children's Health, Beijing, China; ^3^Henan Key Laboratory of Pediatric Inherited & Metabolic Diseases, Henan Children's Hospital, Zhengzhou Hospital of Beijing Children's Hospital, Zhengzhou, China

**Keywords:** whole exome sequencing, copy number variants sequencing, pediatric disorders, cost-effective, clinical utility

## Abstract

**Background:** Both whole exome sequencing and copy number variants sequencing were applied to identify the genetic cause of rare pediatric disorders. In our study, we aimed to investigate the diagnostic yield of parallel tests of trio whole exome sequencing and copy number variants sequencing and its clinical utility.

**Methods:** After collecting detailed clinical information, a total of 60 patients were referred to parallel tests of whole exome sequencing and copy number variants sequencing, which used shared initial libraries.

**Results:** 26 pathogenic or likely pathogenic single nucleotide variants and 11 copy number variants were identified in 32 patients. 65.4% (17/26) of the SNVs were novel. The overall diagnosis rate was 53.3%. For the patients with positive results, 22 (36.7%) patients were diagnosed by whole exome sequencing and 10 (16.7%) patients were diagnosed by copy number variants sequencing. We also reviewed clinical impact on selected cases.

**Conclusion:** We adopted an approach by performing parallel tests of trio whole exome sequencing and copy number variants sequencing with shared initial libraries. This strategy is relatively efficient and cost-effective for the diagnosis of rare pediatric disorders with high heterogeneity.

## Introduction

High genetic heterogeneity in pediatric disorders has been an obstacle to phenotype-based diagnostic testing (Hu et al., 2018). Compared to conventional genetic tests on single-gene scale, tests on genomic scale are more likely to produce higher diagnostic yields and shorter turn-around times (Vissers et al., 2017). Generally, these genomic tests include whole exome sequencing (WES) and chromosomal microarray analysis (CMA). WES has been ordered increasingly in clinical molecular diagnosis laboratories as a powerful tool for rare Mendelian disorders, especially for genetically heterogeneous disorders such as intellectual developmental disorders and multiple congenital anomalies (Bowling et al., 2017; Han and Lee, 2020). According to previous researches, the overall diagnostic yields among different cohorts were ~25% (Yang et al., 2013, 2014; Lee et al., 2014). Recently, multiple researches on clinical utility and cost of WES have provided evidence endorsing it as a first-tier test for children with suspected monogenic disorders (Nguyen and Charlebois, 2015; Monroe et al., 2016; Stark et al., 2016; Hu et al., 2018). CMA has been used as a first-tier clinical diagnostic test in patients with developmental delay (DD)/intellectual disability (ID), autism spectrum disorders (ASD), and multiple congenital anomalies (MCA) since 2010 (Manning et al., 2010; Miller et al., 2010); and copy number variants (CNVs) detected by CMA explained the pathogenesis for over 10% of these cases (Sanmann et al., 2015; Homma et al., 2018; Jang et al., 2019).

Recently, WES or whole genome sequencing (WGS) data were also used to call CNVs through the development of various algorithms and software programs that utilize read-depth information as the main strategy (Wang et al., 2016; Yao et al., 2017). WES-data-based CNV calling is usually performed as a supplement of routine WES test and the accuracy is affected by capture area and amplification efficiency (Rajagopalan et al., 2020; Sun et al., 2020). On the other hand, low-coverage WGS or CNV-seq were widely used in spontaneous miscarriage and prenatal cases, but only a few pediatric CNV-seq testing studies were reported. Most of the studies focused only on neurological disorders (Gao et al., 2019; Jiao et al., 2019). In our study, we performed parallel tests of trio-WES and CNV-seq for 60 children from different clinical departments. Our results showed this parallel testing strategy significantly increased diagnostic yields and lightened the burden of physicians in selecting optimal test.

## Materials and Methods

### Patients

Patients in this research were initially referred to the Beijing Children's Hospital from September 2018 to September 2019, with suspected Mendelian disorders. These patients had DD/ID, ASD, or MCA, and both SNVs and CNVs were highly suspected for their causes of disorders. Next-generation sequencing (NGS) was primarily ordered by the patient's physician and performed by the Laboratory for Genetics of Birth Defects at Beijing Children's Hospital. Written informed consent was obtained from the individuals and their legal guardians for the publication. This study was approved by the Beijing Children's Hospital institutional review board. After recruitment, a total of 60 patients were referred to parallel tests of WES and CNV-seq. The ages of the patients ranged from 1 month to 12 years, and the median age was 1 year. The male-to-female ratio in this study was 1.31:1 (34/26). For the referring reasons, 25 of the patients had multiple congenital anomalies. Ten had DD/ID. Six had the combination of DD/ID and multiple congenital anomalies. Four had autistic traits. The remaining 15 patients had other phenotype, such as congenital heart disease, short stature, recurrent infections, etc. (Supplemental Table 1).

### Whole Exome Sequencing

DNA was isolated from peripheral blood samples obtained from the proband and parents using the Gentra Puregene Blood Kit (QIAGEN, Hilden, Germany). 200-ng genomic DNA of each individual was sheared by Biorupter (Diagenode, Liège, Belgium) to acquire 150~200-bp fragments. The ends of DNA fragment were repaired, and Illumina Adaptors were added (Fast Library Prep Kit, iGeneTech, Beijing, China). After sequencing library was constructed, the whole exome was captured with AIExome Enrichment Kit V1 (iGeneTech, Beijing, China) and sequenced on Illumina NovaSeq 6000 (Illumina, San Diego, USA) with 150 base paired-end reads. Raw reads were filtered to remove low-quality reads using FastQC. Clean reads were mapped to the reference genome GRCh37. Quality control (QC) information included: average read length of >100×, accurate mapping rate of >98%, bases capture rate of >55%, 20× mean depth coverage rate of >96%, duplication rate of <25%, and accurate mapping rate of <96%. Single nucleotide variants (SNVs) were annotated and filtered by TGex (tgex.genecards.org). Variants with a frequency over 1% in the databases of gnomAD, ESP or 1000G were excluded. Variants that lacked segregation in family members were also filtered. Variants were classified following the American College of Medical Genetics and Genomics and the Association for Molecular Pathology interpretation standards and guidelines (Richards et al., 2015).

### Copy Number Variant Calling

To identify large copy number variants (CNVs), part of the library without capture was sequenced directly onto Illumina NovaSeq 6000, and each sample yielded one giga base (Gb) raw data (QC: average read length: >0.3× WGS). An in-house pipeline was applied to map and call CNVs based on the software CNV-seq (Xie and Tammi, 2009; Hu et al., 2019). Clean reads were mapped to the reference genome GRCh37. CNVs called from parental WGS data were used as controls. CNVs reported in multiple peer-reviewed publications or annotated in curated databases as benign or likely benign, CNVs observed frequently in the general population and CNVs containing no genes were filtered. Database of Genomic Variants, DECIPHER database, ClinVar, OMIM, and ClinGen were used for interpretation and classification of the clinical significance of candidate CNVs according to previously reported guidelines (Kearney et al., 2011; Riggs et al., 2019). WES data was also used for CNVs calling, and the samples of the same batch were used as controls. Putative pathogenic or likely pathogenic CNVs identified from low-pass WGS were validated using WES reads depth data.

## Results

### Molecular Diagnosis Rates

We identified 37 pathogenic or likely pathogenic variants in 32 patients. The overall diagnosis rate was 53.3%. For the patients with positive results, 22 (36.7%) individuals were diagnosed by WES (Table 1) and 10 (16.7%) individuals were diagnosed by CNV-seq (Table 2). For patients with MCA and/or DD/ID, the diagnosis rates were all higher than 50%. For patients with autistic traits, only one individual was diagnosed (Table 1).

**Table 1:** Diagnosis rates of patients with different ordering phenotype categories.

**Phenotype**	**All cases**	**WES**	**CNV-seq**	**All positive**	**Positive rate (%)**
MCA	25	10	3	13	52.0
DD/ID	5	3	0	3	60.0
DD/ID+MCA	12	5	5	10	83.3
ASD	4	0	1	1	25.0
Others	14	4	1	5	42.9
Total	60	22 (36.73%)	10 (16.7%)	32	53.3

*ASD, autism spectrum disorders; DD, developmental delay; ID, intellectual disability; MCA, multiple congenital anomalies*.

**Table 2:** Patients with pathogenic and likely pathogenic variants identified by whole exome sequencing.

**Case no**.	**Sex**	**Age (y)**	**Categories**	**Gene**	**Variant**	**Origin**	**Inheritance**	Reported*/novel	**Diagnosed disorders**
4	Male	1	DD/ID+MCA	SMARCA2	c.3593T>G/ p.Val1198Gly	*de novo*	AD	Reported	Nicolaides-Baraitser syndrome
6	Female	0.33	MCA	RNU4ATAC	c.196-570C>T; c.196-605C>T	in-trans	AR	Reported; reported	Roifman syndrome
8	Male	6	Others	PPP2R5D	c.1492C>T/ p.Arg498*	*de novo*	AD	Novel	Mental retardation, autosomal dominant 35
10	Male	5	Others	ELN	c.1621C>T/ p.Arg541*	*de novo*	AD	Novel	Supravalvar aortic stenosis
14	Male	1	Others	DPH1	c.471delC/ p.Arg158fs; c.397T>C/ p.Tyr133His	in-trans	AR	Novel; novel	Developmental delay with short stature, dysmorphic features, and sparse hair
16	Male	0.67	DD/ID	SLC22A5	c.1130T>C/ p.Phe377Ser; c.1400C>G/p.Ser467Cys	in-trans	AR	Novel; reported	Carnitine deficiency, systemic primary
22	Female	3	MCA	MED12	c.6340C>T/ p.Gln2114*	*de novo*	XLR	Novel	Ohdo syndrome
24	Male	5	MCA	CHAF1A	c.1750C>T / p.Arg584*	*de novo*	AD	Novel	novel disorder
27	Male	0.08	MCA	COL1A1	c.4100C>A/ p.Thr1367Asn	Maternal	AD	Novel	Osteogenesis imperfecta
31	Female	3	DD/ID+MCA	NSD1	c.6367_6376delAGTTG TGGGG/ p.Ser2123fs	*de novo*	AD	Novel	Sotos syndrome 1
33	Female	6	DD/ID	KAT6B	c.3663_3664+2delAAGT /p.Asn1222fs	*de novo*	AD	Novel	SBBYSS/Genitopatellar syndrome
35	Male	4	MCA	MID1	c.1863_1879dupGAACTC CATCCACCTCT/ p.Tyr627fs	Maternal	XLR	Novel	Opitz GBBB syndrome, type I
38	Male	0.17	MCA	FGFR2	c.869G>T/ p.Trp290Leu	*de novo*	AD	Reported	Crouzon syndrome
40	Male	0.42	DD/ID	ACAN SETD5	c.7000C>T/ p.Gln 2334*; c.1357C>T/ p.Gln453*	Paternal maternal	AD AD	Novel; novel	Short stature and advanced bone age Mental retardation, autosomal dominant 23
41	Female	1	Others	ELN	c.800-1G>T	Maternal	AD	Reported	Supravalvar aortic stenosis
42	Female	1	MCA	FBN1	c.7180C>T/ p.Arg2394*	Paternal	AD	Reported	Marfan syndrome
46	Male	3	DD/ID+MCA	ATRX	c.6254G>A/ p.Arg2085His	Maternal	XLR	Reported	Alpha-thalassemia/mental retardation syndrome
47	Male	1	DD/ID+MCA	ANKRD11	c.1681G>T/ p.Glu561*	*de novo*	AD	Novel	KBG syndrome
49	Male	0.42	MCA	PIK3CA	c.2740G>A/ p.Gly914Arg	*de novo*	AD	Reported	Megalencephaly-capillary malformation
50	Male	12	MCA	FBN1	c.1995C>A/ p.Tyr665*	*de novo*	AD	Novel	Marfan syndrome
54	Male	0.25	DD/ID+MCA	SOX9	c.340G>T/ p.Val114Leu	*de novo*	AD	Novel	Campomelic dysplasia
58	Male	0.92	MCA	CREBBP	c.3306T>A/ p.Tyr1102*	*de novo*	AD	Novel	Rubinstein-Taybi syndrome

DD, developmental delay; ID, intellectual disability; MCA, multiple congenital anomalies; *Variants included in public databases or previously published literature.

### Characterization of Variants

For the 37 pathogenic or likely pathogenic variants, 26 were SNVs and 11 were CNVs (Tables 2, 3). Twenty-six SNVs were located in 21 different genes, and most of them (17/26, 65.4%) were in autosomal dominant diseases genes. Six variants were located in three autosomal recessive diseases genes, and three variants were located in X-linked recessive diseases genes. 65.4% (17/26) SNVs were not reported in previous literature or public population databases. Thirteen variants were identified to be *de novo*, six variants were compound heterozygous inherited from the parents, five were inherited from parents with similar phenotypes, and two X-linked recessive genes variants inherited from maternal carriers. Mutation in only two genes, *ELN* and *FBN1*, occurred in more than one patient, indicating the high heterogeneity of pediatric disorders (Table 2). For 11 CNVs identified by CNV-seq, the sizes ranged from 1.41 to 43.68 Mb. All these variants were also called from WES data to eliminate false positive variants. All these variants were *de novo* (Table 3). Patient 23 had a distal deletion and distal duplication, which was potentially inherited from a parent with balanced translocation. Further karyotype test may help to verify this hypothesis.

**Table 3:** Patients with pathogenic and likely pathogenic variants identified by copy number variation sequencing.

**Case no**.	**Sex**	**Age (y)**	**Categories**	**CNV**	**CNV length**	**Diagnosed disorders**
1	Male	0.17	MCA	seq[GRCh37]del(22)(22q11.2)chr22:g.18963217_21541547del	2.58 Mb	22q11.2 deletion syndrome
3	Female	1	MCA	seq[GRCh37]del(5)(5p15.33p15.1)chr5:g.54215_16752126del	16.70 Mb	Cri-du-chat syndrome
11	Female	0.58	DD/ID+MCA	seq[GRCh37]del(9)(9p24.3p11.2)chr9:g.152081_43829455del	43.68 Mb	9p deletion syndrome
13	Male	8	ASD	seq[GRCh37]del(22)(22q11.21)chr22:g.19028192_21466206del	2.44 Mb	22q11.2 deletion syndrome
20	Male	4	DD/ID+MCA	seq[GRCh37]del(11)(11q24.2q25)chr11: g.124175265_134974797del	10.80 Mb	NA
23	female	0.17	DD/ID+MCA	seq[GRCh37]dup(8)(8q24.3)chr8: g.141319205_144918800dup; seq[GRCh37]del(18)(18q22.2q23)chr18: g.67971593_77882168del	3.60 Mb; 9.91 Mb	NA
28	Female	8	DD/ID+MCA	seq[GRCh37]del(7)(7q11.23)chr7:g.72645343_74161920del	1.52 Mb	Williams-Beuren syndrome
29	Female	15	MCA	seq[GRCh37]del(14)(14q13.1q21.1)chr14: g.35268524_38367321del	3.10 Mb	NA
36	Male	3	DD/ID+MCA	seq[GRCh37]del(4)(4q25q26)chr4:g.111697561_116176319del	4.48 Mb	NA
59	Female	0.67	MCA	seq[GRCh37]del(22)(22q11.2)chr22:g.18905953_20318038del	1.41 Mb	22q11.2 deletion syndrome

*ASD, autism spectrum disorders; DD, developmental delay; ID, intellectual disability; MCA, multiple congenital anomalies*.

### Turn-Around Time (TAT) and Cost Analysis

We aimed to decrease the difficulty for physicians to select optimal test between WES and CNV-seq, and to shorten TAT, as well, by parallel testing. We tracked the TAT of our parallel test for these patients; it ranged from 38 days to 125 days. The median TAT is 72 days with 69.2% of patients received reports within 80 days. The raw data were usually obtained within 20 days. Initial test results were generated 10–20 days afterwards and were delivered to ordering physicians to further check potential phenotype. Typically, negative cases cost more days because multiple rounds of communication with ordering physicians would be guaranteed, and a second analysis by another geneticist was performed before the formal reports were issued.

The direct cost of running a parallel trio-WES and CNV-seq is about $600 US dollars ($200 per person), including library construction (~$100 USD), WES (~$80 USD, 100×) and low-coverage WGS (~$10 USD, 0.3×), and Sanger validation (~$10 USD). These results showed that our parallel test strategy was affordable and additional CNV-seq did not increase the prime cost since the generation of one Gb raw data usually costed < $20 USD.

### Clinical Impact of Genetic Diagnoses

Over half of the patients ended their diagnosis odysseys after parallel tests. The positive diagnostic results affected clinical management including appropriate genetic counseling, other systemic evaluation, and change of treatment. We provided examples of impacts on patients' clinical managements, below.

Case 4 was a 12-month-old boy. He was referred to Clinic of Development & Behavioral Pediatrics for Short Stature and DD. A *de novo SMARCA2* mutation was identified and was therefore diagnosed as Nicolaides-Baraitser syndrome (Zhang P. et al., 2019). This syndrome was less recognizable and always misdiagnosed as Coffin-Siris syndrome, Williams syndrome, etc. Once molecular diagnosis was confirmed, this patient was referred to neurologist for seizure evaluation. Ophthalmological and audiological examinations were also ordered.

Case 40 was a 5-month-old boy who had developmental and growth delays. Mutations in two genes were identified. DD was caused by *SETD5* mutation inherited from his mother with intellectual disability and growth delay was caused by *ACAN* mutation inherited from his father with short stature. Referral to an early intervention program is recommended for access to occupational, physical, speech, and feeding therapy. Growth pattern should be monitored and growth hormone therapy can improve height increase (Hu et al., 2017).

Case 29 was a 15-year-old girl. She was first referred to Department of Respiration for Pulmonary Lesions. Further evaluation revealed short stature, ataxia, tooth agenesis, depigmentation/hyperpigmentation of skin and absence of secondary sex characteristics. Parallel tests identified a 3.1-Mb *de novo* interstitial deletion of the 14q13.2q21.1 region encompassing 17 OMIM genes (Hu et al., 2019). In these genes, *NKX2*-1 deletion is responsible for choreoathetosis, hypothyroidism, and neonatal respiratory distress and haploinsufficiency of *PAX9* causes oligodontia phenotype (Das et al., 2002; Santen et al., 2012; Hayashi et al., 2015). For this patient, thyroid function testing should be performed annually, and when hypothyroidism is discovered, thyroid hormone replacement therapy should be initiated. For choreoathetosis, Tetrabenazine and Levodopa therapy have been reported to effectively reduce chorea (Setter et al., 2009; Rosati et al., 2015).

Cases 1, 13, and 59 all had 22q11.2 deletion syndrome. This was the only recurrent CNV disorder in our cohort. Case 1 was initially considered as DiGeorge syndrome for the characteristic facial features. Case 13 was referred only for autistic behaviors, and Case 59 was referred for congenital heart disease and laryngomalacia. After diagnosis, these patients took further evaluation and treatment following the clinical practice guidelines for individuals with 22q11.2 deletion syndrome (Bassett et al., 2011).

## Discussion

In this study, we performed parallel tests of WES and CNV-seq for 60 patients. Thirty-seven pathogenic or likely pathogenic variants in 32 patients were identified, and the overall diagnosis rate was 53.3%. Our study provided preliminary results of clinical utility of parallel tests of CNVs and SNVs at the same time in pediatric patients with developmental delay/intellectual disability, autism spectrum disorders, and multiple congenital anomalies.

Over 6500 phenotypes were included in the OMIM by the end of 2019 and most of them were onset during childhood. Genetic pediatric disorders are highly heterogeneous and relatively rare, which, for investigating them, necessitates a process of serial testing for specific conditions (Han and Lee, 2020). In consequence, this strategy may be expensive and time-consuming. The molecular diagnosis of these rare diseases is based on the appropriate choice among tests of karyotype, Sanger sequencing, multiplex ligation dependent probe amplification, CMA, NGS, etc., which requires a physician with medical genetics training and experience. However, most children's hospitals do not have independent genetic clinic for patients with genetic disorders (Hu et al., 2018). Meanwhile, a more comprehensive strategy of genomic-first approach should be applied to achieve higher positive rate and reduce diagnostic odyssey (Johnson, 2015).

Previous WES studies revealed a diagnosis rate of ~25–30% in nonselective patients (Yang et al., 2013, 2014; Lee et al., 2014; Daoud et al., 2016; Retterer et al., 2016). Moreover, additional pathogenic and likely pathogenic CNVs were also identified in over 10% of patients (Sanmann et al., 2015; Homma et al., 2018; Jang et al., 2019). The development of NGS data based CNV calling algorithm makes it possible to identify SNVs and CNVs at same time. In this study, we performed trio-WES and low-coverage WGS (0.3×) simultaneously and uncovered pathological causes in over half of the patients. The rate of patients with neurological features was 67% (14/21), which was higher than previous reports (Gao et al., 2019; Jiao et al., 2019). However, it was notable that our sample size was relatively small, and more researches were needed to better the clinical efficacy.

The application of NGS to detect genome-wide CNVs is a recently developed method (Xie and Tammi, 2009). Comparing to pediatric disorders, CNV-seq was more widely used in prenatal screening and diagnosis, which required lower resolution (Liang et al., 2014; Zhang R. et al., 2019; Zhao and Fu, 2019). The resolution of CNV-seq can be adjusted by increasing or decreasing the sequencing data volume of raw data. For pediatric diagnosis, the resolution was usually >100 Kb (Gao et al., 2019; Jiao et al., 2019). By comparing differences of aligned reads number between case and control samples, losses or gains of chromosomal regions can be identified and quantitated. Therefore, it has been reported that it is easier to identify low-level mosaicism by CNV-seq, rather than CMA (Grotta et al., 2015). Furthermore, identifying CNVs by sequencing has other advantages, including lower starting input of the DNA, cheaper cost, shorter TAT and higher throughput.

In the present study, our parallel tests of WES and low-pass WGS (0.3×)-based CNV-seq decreased TAT and human labor without a significantly increased cost. Compared to WES, the extra cost was used for WGS of each sample to yield one Gb raw data directly, using the same sample library constructed during WES before capture, and the sequencing cost for 1 Gb raw data was about $10 USD with Illumina NovaSeq, in this research. We identified pathogenic or likely pathogenic variants with WES and CNV-seq in 36.7 and 16.7% of patients, respectively, which was comparable with previous study, indicating that our strategy is efficient to simultaneously identify SNVs and CNVs. Therefore, the ordering physicians do not need to choose between CMA and NGS, a choice which is dependent on clinical expertise, and they are more likely to identify clinically relevant variants in patients with atypical presentation or novel phenotypes. The mean age of positive cases at diagnosis was 3 years (95% CI: 1.73, 4.26). Early diagnosis avoided long diagnostic odyssey once symptoms appear, but also provided opportunities for early intervention, helping to improve outcomes and prolonging life.

In conclusion, our study provided preliminary experience of parallel tests of WES and CNV-seq with same initial library and evaluated the clinical utility. Compared to traditional trio-WES test, our strategy increased the diagnosis rate from 36.7 to 53.3% in our relatively small cohort. With less depending on physicians' selection between SNVs and CNVs tests, we propose this strategy is efficient and cost-effective for the diagnosis of genetic pediatric disorders with high heterogeneity.

## Data Availability Statement

The datasets for this article are not publicly available due to concerns regarding participant/patient anonymity. Requests to access the datasets should be directed to the corresponding author.

## Ethics Statement

Written informed consent was obtained from the individuals and their legal guardians for the publication.

## Author Contributions

XH, RG, JG, and ZQ collected the clinical data. CH and WL designed the study. XH, RG, and JG performed data analysis. XH and CH wrote the manuscript. All authors contributed to data acquisition and data interpretation, critically revised the manuscript for important intellectual content, approved the final, submitted version of the manuscript, and agreed to be accountable for all aspects of the work.

## Conflict of Interest

The authors declare that the research was conducted in the absence of any commercial or financial relationships that could be construed as a potential conflict of interest.

## Abbreviations

WES: whole exome sequencing
CNV-seq: copy number variants sequencing
SNVs: single nucleotide variants
CNVs: copy number variants
CMA: chromosomal microarray analysis
DD: developmental delay
ID: intellectual disability
ASD: autism spectrum disorders
MCA: multiple congenital anomalies
WGS: whole genome sequencing
NGS: next-generation sequencing
TAT: turn-around time
QC: quality control.
